# Programme quality indicators of HIV drug resistance among adolescents in urban versus rural settings of the centre region of Cameroon

**DOI:** 10.1186/s12981-020-00270-7

**Published:** 2020-05-12

**Authors:** Joseph Fokam, Armanda Nangmo, Carlson Wandum, Desire Takou, Maria Mercedes Santoro, Anne-Esther Njom Nlend, Francis Ndongo Ateba, Paul Koki Ndombo, Nelly Kamgaing, Cedric Kamta, Andre Essiane, Virginie Lambo, Charles Fokunang, Dora Mbanya, Vittorio Colizzi, Carlo-Federico Perno, Alexis Ndjolo

**Affiliations:** 1 Virology Laboratory, Chantal BIYA International Reference Centre for Research on HIV/AIDS Prevention and Management (CIRCB), P.O BOX: 3077, Messa, Yaoundé, Cameroon; 2 Faculty of Medicine and Biomedical Sciences (FMSB), University of Yaoundé I, Yaoundé, Cameroon; 3 National HIV Drug Resistance Working Group (HIVDRWG), Ministry of Public Health, Yaoundé, Cameroon; 4 Faculty of Health Sciences (FHS), University of Bamenda, Bambili, Cameroon; 5 University of Rome Tor Vergata (UTV), Rome, Italy; 6 National Social Welfare Hospital (NSWFH), Yaoundé, Cameroon; 7 Mother–Child Centre of the Chantal BIYA’s Foundation (MCC-CBF), Yaoundé, Cameroon; 8 University Teaching Hospital (UTH), Yaoundé, Cameroon; 9 University of Milan (UM), Milan, Italy; 10 Mfou District Hospital (MDH), Mfou, Cameroon; 11 Mbalmayo District Hospital (MDH), Mbalmayo, Cameroon; 12 Nkomo Medical Center (NMC), Nkomo, Cameroon

**Keywords:** Programme quality indicators, HIV drug resistance, Antiretroviral treatment, Adolescents, Cameroon

## Abstract

**Background:**

The high rate of mortality among HIV-vertically infected adolescents might be favoured by HIV drug resistance (HIVDR) emergence, which calls for timeous actions in this underserved population. We thus sought to evaluate program quality indicators (PQIs) of HIVDR among HIV-vertically infected adolescents on antiretroviral therapy (ART).

**Methods:**

A study was conducted in the Centre region of Cameroon among adolescents (10–19 years) receiving ART in two urban (The Mother–Child Centre of the Chantal BIYA Foundation, the National Social Welfare Hospital) and three rural (Mfou District Hospital, Mbalmayo District Hospital and Nkomo Medical Center) health facilities. Following an exhaustive sampling from ART registers, patient medical files and pharmacy records, data was abstracted for seven PQIs: *on*-*time drug pick*-*up*; *retention in care; pharmacy stock outs*; *dispensing practices*; *viral load coverage*; *viral suppression and adequate switch to second*-*line*. Performance in PQIs was interpreted following the WHO-recommended thresholds (desirable, fair and/or poor); with p < 0.05 considered significant.

**Results:**

Among 967 adolescents (888 urban versus 79 rural) registered in the study sites, validated data was available for 633 (554 in urban and 79 in rural). Performance in the urban vs. rural settings was respectively: *on*-*time drug pick*-*up* was significantly poorer in rural (79% vs. 46%, p = 0.00000006); *retention in care* was fair in urban (80% vs. 72%, p = 0.17); *pharmacy stock outs* was significantly higher in urban settings (92% vs. 50%, p = 0.004); *dispensing practices* was desirable (100% vs. 100%, p = 1.000); *viral load coverage* was desirable only in urban sites (84% vs. 37%, p < 0.0001); *viral suppression* was poor (33% vs. 53%, p = 0.08); *adequate switch to second*-*line* varied (38.1% vs. 100%, p = 0.384).

**Conclusion:**

Among adolescents on ART in Cameroon, dispensing practices are appropriate, while adherence to ART program and viral load coverage are better in urban settings. However, in both urban and rural settings, pharmacy stock outs, poor viral suppression and inadequate switch to second-line among adolescents require corrective public-health actions to limit HIVDR and to improve transition towards adult care in countries sharing similar programmatic features.

## Introduction

There is a remarkable reduction in Human immunodeficiency virus (HIV) associated mortality (1.1 million [940,000–1.3 million] in 2015, with a 45% decline since 2005 [1]) due to the roll-out of antiretroviral therapy (ART) in resource-limited settings (RLS), especially those with the highest burden of HIV [2]. Among these RLS, sub-Saharan Africa (SSA) is the most affected with ~ 70% out of the 37.9 million people infected worldwide [3]. Furthermore, SSA consists of ~ 80% of 1.8 million adolescents aged 10–19 years living with perinatal or behaviourally acquired HIV infection [4]. Of note, in spite of the overall decreasing mortality, AIDS remains the leading cause of death among adolescents in SSA [5]. This is particularly true for adolescents who acquire HIV as babies and survive to teen age [5].

Since 2010, AIDS related deaths have reduced by half in the general population, as compared to only 5% among adolescents [6, 7]. As HIV infected children are gradually growing towards adolescence, adequate measures are of paramount importance to keep them alive and to ensure a successful transitioning from paediatrics to adult care [4, 7]. Besides identifying the most suitable ART regimens for adolescents, understanding challenges faced by these adolescents is concerning [7, 8]. Such challenges might be due to individual, program and viral factors, all contributing to ART failure, emergence of HIV drug resistance (HIVDR), limited ART options and finally continuous mortality of this vulnerable and underserved population [8].

As HIVDR is a major concern in the era of global ART scale-up, the use of ARV drugs with a low-genetic barrier to resistance for treating HIV vertically infected adolescents in SSA suggests higher risk of virologic failure [9]. This phenomenon becomes even more appealing in the frame of limited access to reference ART monitoring in routine practice, due to cost and lack of molecular diagnostic facilities [9]. Thus, surveillance of HIVDR among adolescents would be useful in addressing preventable HIVDR and implement corrective actions in RLS such as Cameroon [10, 11].

Cameroon is a country located in SSA with a population of about 23.34 million [12], with an estimated 520,069 people living with HIV and among whom 51.71% (268,939) were receiving ART by the end of June 2018 (National AIDS report bulletin, September 2018). The epidemiological burden of HIV in Cameroon is decreasing (from 4.3% in 2011 to 3.4% in 2018) [13]. At a country-level, this progress is largely due to efforts in implementing Prevention of Mother-To-Child Transmission (PMTCT) option B+ since 2014, the “Test and Treat” strategy since 2016, HIV early infant diagnosis since 2007, increasing viral load coverage, as well as monitoring HIVDR programme quality indicators (PQI), previously referred to as early warning indicators (EWI) of HIVDR [14–16]. These indicators entail risks to HIVDR emergence that include programme factors (dispensing practices of mono- or dual-therapy, pharmacy stock outs, viral load coverage, adequate switch to second-line ART); patient factors (retention in care, on-time drug pick-up by patients); and viral factors (viral load suppression below 1000 RNA copies/ml) [14–16].

Studies on the aforementioned patient, programmatic and viral factors carried out among paediatric populations (age group ≤ 14 years) and adult populations (age group ≥ 15) might not clearly unveil factors specific to the target population of HIV-infected adolescents, especially those with vertical infection who have a peculiar long-term ART experience and a high likelihood of infrequent adherence [2]. Thus, considering challenges in monitoring immunological and virologic parameters, as well as individual HIVDR testing among adolescents in RLS like Cameroon, it is hence essential to evaluate the HIVDR PQIs among vertically infected adolescents on ART and identify driving factors of poor ART outcomes in this target population [16, 17]. We thus aimed at evaluating HIVDR PQI among adolescents in the centre region of Cameroon, and to compare the performance of HIVDR PQI between urban and rural settings.

## Methods

### Study design and target population

As baseline assessment within the *Resistance Evolution among Adolescents in Yaoundé and its surroundings* (*READY*-*study*), a retrospective study was conducted in May 2018 to evaluate HIVDR PQIs among HIV vertically infected adolescents aged 10–19 years and receiving ART in one of the chosen five ART sites of the centre region of Cameroon, with participants enrolled following an exhaustive sampling.

### Description of study settings

Two urban health facilities (The Mother and Child Centre [MCC] of the Chantal BIYA foundation and the National Social Welfare Hospital [NSWH], both in Yaoundé) and three rural health facilities (the Mfou District Hospital [MFDH], the Mbalmayo District Hospital [MBDH] and the Nkomo Medical Centre [NMC]) were enrolled in the study. Urban sites were reference centres specialised on paediatric care, while rural sites were comprehensive healthcare centres having HIV management units and located 30–50 km from the urban settings. Regarding the level of healthcare facilities, the two urban sites belonged to secondary structures and the three rural sites to tertiary structures; all sites were under the public sector apart from the NSWH that was para-public; first- and second-line ART regimens were provided in all sites. A monitoring committee (consultative body for ART monitoring and retention in care), consisting of clinicians, pharmacists/clerks, medical biologists/technicians, nurses, counsellors), was present in urban sites. ART registers and patient medical files were available in all sites but only urban sites had an electronic database system.

### Sampling strategy

Sites were selected based on the following criteria: (a) years of experience in paediatric ART management (≥ 3 years); (b) availability of first and second-line ART regimens at the hospital pharmacy; (c) availability of national ART guidelines; (d) availability of ART registers; patient medical files and/or a database; (e) the number of adolescents on ART per site (n ≥ 15); and geographical location (urban versus rural).

A non-probabilistic and exhaustive sampling method was used on available data of HIV infected adolescents receiving ART during the reporting period: from 1st February 2017 through 31st January 2018, with 3 additional months to monitor defaulters or those lost to follow-up.

In each of the five study sites, adolescents on ART were enrolled consecutively until full sampling at the study site. The minimal sample size required to carry out this study was established following World Health Organisation (WHO) recommended strategy as indicated [18].

### Data collection, quality assurance and data validation

Data was collected from ART registers, patient medical files, pharmacy records, and then abstracted into Early Warning Indicators (EWI) data sheets [18]. Seven HIVDR PQIs were assessed during the study-reporting period as follows: PQI-1 (*On time drug pick*-*up*), PQI-2 (*Retention in care*), PQI-3 (*Pharmacy stock outs*), PQI-4 (*Dispensing practices*), PQI-5 (*Viral load coverage*), PQI-6 (*Viral load suppression*), and PQI-7 (*Adequate switch to second*-*line ART*). Briefly, PQI-1 and PQI-4 required a cross-sectional data abstraction following a consecutive enrolment, while the other PQIs (2, 3, 5, 6 and 7) were abstracted following a longitudinal approach.

Incoherent data was resolved by retrieving additional source documents available at the clinic. HIVDR PQIs were defined according to the WHO sampling strategy to ensure representativeness of each site [16, 19]. To ensure reliability in collected data, two final year medical students were trained on HIVDR, PQI and ART programme, as well as on the procedures for PQI data abstraction following the WHO strategy [17–20]. Abstracted data was crosschecked for consistency, then data validation was done by a supervisor (senior virologist) with field experience on the collection, monitoring, evaluation and reporting of data in the ART program.

### Data analysis

Validated data was analysed according to definition of the WHO HIVDR PQI and their respective target indicators (Table 1) [11]. Performance in PQIs was interpreted following the WHO-recommended thresholds (desirable, fair and/or poor), and then compared between urban vs. rural settings, taking p-values < 0.05 as statistically significant.

**Table 1:** Quality indicators definition and their respective site performance levels [11]

PQI title	Definition	Numerator	Denominator	Performance
PQI-1 On-time ARV drug pick-up	Percentage of adolescents that pick-up ART no more than 2 days late at the first pick-up after the baseline pick-up.	Number of adolescents picking-up their ART “on time” at the first drug pick-up after baseline pick-up date	Number of adolescents who picked-up ARV drugs on or after the designated PQI sample start date	The desirable performance (green): > 90%, Fair performance (amber): 80–90%, Poor performance (red): < 80%
PQI-2 Retention in care	Percentage of adolescents known to be alive and on ART 12 months after enrolment	Number of adolescents who are still alive and on ART 12 months after enrolment	Total number of adolescents (excluding transfers out) who were expected to achieve 12-month outcomes	Desirable performance (green): > 85% Fair performance (amber): 75–85% Poor performance (red): < 75%
PQI-3 Antiretroviral drug stock outs	Percentage of antiretroviral drug stock-outs during a 12-month period	Number of months in the year in which there were ARV drug stock outages for any ARVs in any of the standard ART regimens supplied by the site or the pharmacy at which the site’s patients pick up ARV drugs	12 months	The desirable performance: Zero (0%)
PQI-4 Dispensing Practices	Percentage of adult patients picking up mono or dual ARV therapy	Number of adolescents who pick up from the pharmacy, a regimen consisting of one or two ARVs	Number of adolescents picking up ART on or after the designated EWI sample start date	The desirable performance (green): 0%; Poor performance (red): > 0%
PQI-5 Viral load coverage	Number of patients who by national policy should have received a 12-month viral load test, by clinic	Number of adolescents who have a viral load performed at 12 months after enrolment	Total number of adolescents on ART on site (excluding transfers out) 12 months after enrolment	Desirable performance (green): ≥ 70% Poor performance (red): < 70%
PQI-6 Viral load suppression	Percentage of adolescents receiving ART of ART whose viral load is < 1000 copies/ml 12 months after enrolment	Number of adolescents receiving ART 12 months after study enrolment and whose viral load is < 1000 copies/ml	Number of adolescents who have a viral load performed at 12 months after enrolment	Desirable performance (green): > 90%, Fair performance (amber): 70–90%, Poor performance (red): < 70%
PQI-7 Adequate switch to second-line antiretroviral therapy regimens	Percentage of adolescents on ART on site with a change of therapy from first line ART to second line ART based on viral load result	Number of adolescents on second line ART on site after 12 months	Number of adolescents on ART on site after 12 months	The desirable performance: 100% of people switch to second-line antiretroviral therapy guided by a viral load result

ARV: antiretroviral; ART: Antiretroviral therapy; PQI: programme quality indicator

Aggregated data of site performance was classified as: “very unsatisfactory” (if 0% of sites met an acceptable performance), “unsatisfactory” (if 0–49% of sites met an acceptable performance), “satisfactory” (if 50–79% of sites met an acceptable performance), or “excellent” (if ≥ 80% of sites met an acceptable performance).

## Results

### Socio-demographic characteristics of study participants

At the beginning of the study, 888 adolescents were receiving ART in the two urban sites, and electronic software for routine ART monitoring was available only at the NSWH. In the rural sites, a total of 79 adolescents were receiving ART and were entirely enrolled. According to geographical location, females were dominant (55.9%) in the urban sites while males represented the majority (52.3%) in rural sites; the median [IQR] age of adolescents was 15 [13–18] years in urban vs. 12 [11–17] years in rural sites; and the overall patient/staff ratio was 131/1 (from 73/1 at MCC to 222/1 at MBDH), as detailed in Table 2. According to geographical location, patient/staff ratio was seemingly acceptable (ratio of 103 patients for 1 staff) in urban as compared to (ratio of 150 patients for 1 staff) in rural settings.

**Table 2:** Patient to staff ratio

Site	Mother–Child Center	National Social Welfare Hospital	Mfou district Hospital	Mbalmayo district Hospital	Nkomo medicalised Center
Active file	1335	4128	1030	1330	1043
Number of adolescents	667	221	18	31	30
Number of doctors	3	10	1	1	2
Number of nurses	4	5	2	1	2
Number of pharmacy attendants	2	1	1	1	1
Number of laboratory technicians	11	10	0	0	4
Number of psychosocial agents	17	15	3	3	4
Patient to staff ratio	73:1	133:1	147:1	222:1	80:1

### Performance of HIVDR programme quality indicators by geographical location

PQI-1 (*On time drug pick*-*up*): Overall, none of the study sites met the desirable performance (≥ 90%) for timely pick-up of ARV drugs as per pharmacy appointments. Nonetheless, one site (from the urban setting) had a fair performance (81.7%). Interestingly, aggregated data by geographical location revealed that urban sites had a significantly better performance (p = 0.00000006) as compared to rural sites (Table 3).

**Table 3:**
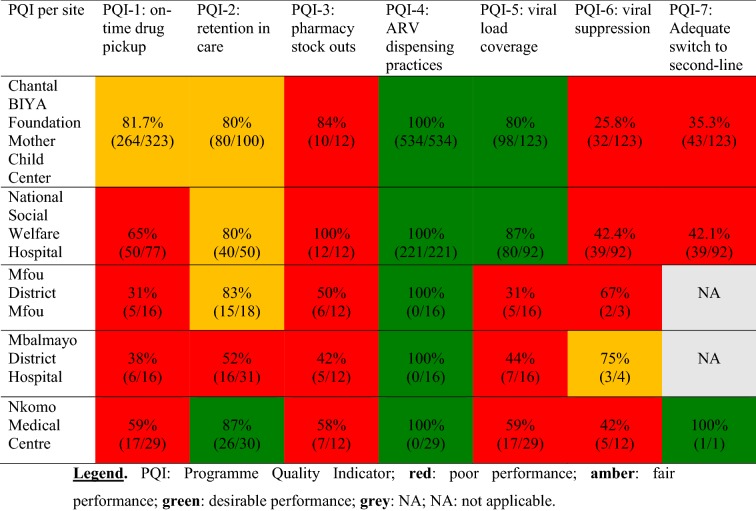
Summary of site performance for each programme quality indicator

PQI, Programme quality indicator; red, poor performance; amber, fair performance; green, desirable performance; grey, NA; NA, not applicable

PQI-2 (*Retention in care*): Overall, only one study site (from the rural setting) met the desirable performance (≥ 85%) for adolescent retention in care at 12 months after ART initiation (Table 3). Remarkably, aggregated data by geographical location revealed that both urban sites reached a fair performance (80%) versus a non-significant poorer performance (72%) in rural sites; p = 0.17. Major causes of poor retention in care after one year were loss to follow-up, deaths, and voluntary ART interruption.

PQI-3 (*Pharmacy stock outs*): Overall, none of the sites met the performance of zero drug stock outs during the 12-month reporting period (Table 3). Curiously, aggregated data by geographical location revealed a significantly higher occurrence of drug stock outs in urban as compared to rural settings (92% vs. 50%, p = 0.004). Thus, drug formulations received by adolescents were not continuously available at drug refill at the level of the pharmacy.

PQI-4 (*Dispensing practices*): Overall, the five study sites reached the desired target performance of 100% good dispensing practices (Table 3). Thus, all the health facilities prescribed a triple ART to all adolescents both in urban and rural sites (p = 1.00). This revealed good prescribing and dispensing practices of ARV drugs according to national ART guidelines.

PQI-5 (Viral load coverage): Overall, only urban sites (MCC and NSWH) met the desirable target for viral load coverage (84%) as compared to significantly poor performance in rural sites (37%); p < 0.0001). Of note, viral load coverage in rural sites ranged from 31 to 59%, a performance very far from the minimal threshold of 70% required assessment (Table 3).

PQI-6 (Viral suppression): Overall, none of the sites met the minimal target performance (≥ 70%) for viral load suppression (Table 3). Interestingly, aggregated data by geographical location revealed that urban settings have lower performance as compared to rural settings (33% vs. 53% respectively, p = 0.08). Interpreting this finding is limited by low sampling in rural sites.

PQI-7 (*Adequate switch to second*-*line*): Overall, one site (from the rural setting) met the target performance (100%) for adequate switch to second-line ART based on a viral load result (Table 3). Remarkably, aggregated data by geographical location revealed varied performance between urban and rural, though non-significantly (38.1% vs. 100%, respectively, p = 0.384).

### Overall site performance of HIVDR programme quality indicators

- PQI-1 (*On*-*time drug pick*-*up*) revealed only a fair performance at one site (the Mother Child Center [MCC]), giving an acceptable site performance of 20% (1/5) in the entire study. Thus, site performance for timely drug pick-up among adolescents was “unsatisfactory”.
- PQI-2 (*Retention in care*) revealed a desirable performance at one site (Nkomo Health Centre [NHC]) and a fair performance at three other sites, giving an acceptable site performance of 80% (4/5) in the entire study. Thus, site performance for retention in care of adolescents was “excellent”.
- PQI-3 (*Pharmacy stock*-*outs*) revealed a poor performance in all the five sites, giving a site performance of 0% (0/5) in the entire study. Thus, site performance for drug stock outs was “very unsatisfactory”.
- PQI-4 (*Dispensing practices*) revealed a desirable performance in all the five study sites, giving an acceptable site performance of 100% (5/5) in the entire study. Thus, site performance for ARV dispensing practices to adolescents was “excellent”.
- PQI-5 (*Viral load coverage*) revealed a desirable performance in two study sites, giving an acceptable site performance of 40% (2/5) in the entire study. Thus, site performance for viral load coverage for adolescents was “unsatisfactory”.
- PQI-6 (*Viral suppression*) revealed only a fair performance at one study site, giving an acceptable site performance of 20% (1/5) in the entire study. Thus, site performance for viral load suppression among adolescents was “unsatisfactory”.
- PQI-7 (*Adequate switch to second line*) revealed a desirable performance in one of the three study sites eligible for this assessment, giving an acceptable site performance of 33.3% (1/3) in the entire study. Thus, site performance for switching to second-line based on a viral load result among adolescents was “unsatisfactory”.

Overall, site performance of these seven PQIs classified *pharmacy stock outs* as the most challenging indicator (very unsatisfactory); followed by *on*-*time drug pick*-*up*, *viral load suppression*, *adequate switch to second*-*line ART* and *viral load coverage* (all being unsatisfactory). Targets on *retention in care* and *dispensing practices* were excellent.

### Site performance according to patient-staff ratio

Using a patient-staff ratio of 100/1 as “normal workload” vs. > 100/1 as “heavy workload”, acceptable site performance of PQI was as follows:

- PQI-1 (*On*-*time drug pick*-*up*)—sites with “normal workload” performed better as compared to those with “heavy workload”: 50% (1/2) vs. 0% (0/3) respectively, p = 0.400;
- PQI-2 (*Retention in care*)—sites with “normal workload” performed better as compared to those with “heavy workload”: 100% (2/2) “normal workload” vs. 67% (2/3) “heavy workload” respectively, p = 1.000;
- PQI-3 (*Pharmacy stock*-*outs*)—Comparison with workload was not applicable due to poor performance in all sites;
- PQI-4 (*Dispensing practices*)—Comparison with workload was not applicable due to excellent performance in all sites;
- PQI-5 (*Viral load coverage*)—sites with “normal workload” performed better as compared to those with “heavy workload”: 50% (1/2) “normal workload” vs. 33% (1/3) “heavy workload” respectively, p = 1.000;
- PQI-6 (*Viral suppression*)—sites with “normal workload” performed less as compared to sites with “heavy workload”: 0% (0/2) “normal workload” vs. 33% (1/3) “heavy workload” respectively, p = 1.000;
- PQI-7 (*Adequate switch to second line*)—sites with “normal workload” performed less as compared to sites with “heavy workload”: 0% (0/2) “normal workload” vs. 100% (1/1) “heavy workload” respectively, p = 0.333.

Overall, sites with heavy workload appeared with suboptimal performance for *on*-*time drug pickup, retention in care, and viral load coverage*, thus indicating heavy workload as a potential risk factor of poor monitoring of adolescents receiving ART.

## Discussion

In these clinics from the Cameroonian context, the majority of adolescents receiving ART are monitored in urban health facilities (~ 90%). Interestingly, one urban clinic alone (The Mother and Child Centre [MCC] of the Chantal BIYA Foundation) enrols more than half of these adolescents. Of note, this is a specialised paediatric centre for children and adolescents whereas the other urban clinic (National Social Welfare Hospital—NSWH) is specialised in both paediatric and adult populations. Thus, this accounts for the small number of adolescents found in NSWH despite their huge active file. Furthermore, there are fewer number of adolescents managed in rural health facilities, this is partly due to referral of some cases to reference centres (often based in urban settings). The overall patient-staff ratio (131/1) indicates a heavy workload in health facilities managing patients on ART, especially those clinics located in rural settings [21]. Adolescents were older in urban settings (15 vs. 12 years), and this reflects the earlier launching of ART programmes in urban settings [18, 19].

Regarding *on*-*time drug pick*-*up*, only one site (from the urban setting) had an acceptable performance, and rural sites experienced a significantly lower performance for patients refilling their drugs on time. These findings are similar to those obtained in adult populations in Cameroon [18, 19, 22] and in other settings [17, 23–27]. In the rural areas, this delay could be due to the fact that patients live at far distances from the health facilities and have difficult transportation systems [18, 19]. Of note, within the Namibian ART programme, 82% of paediatric HIV clinics which attained a desired performance for timely drug pick-up were essentially those in the reference sites than those in the outreached facilities [20]. The poor outcome recorded in our study might be due to adherence challenges during adolescence in spite of the counselling sessions. This therefore calls for additional strategies to minimize missed appointments and to improve adherence. Similarly, 80.6% of paediatrics sites in Zimbabwe also had a poor performance [24]. In this frame, non-disclosure of HIV status during childhood plays a major role as counselling sessions are held mainly with parents/guardians. Thus, setting-up adolescent peer support groups would be relevant [17]. On-time drug pick-up was better in sites with “normal workload” as compared to those with “heavy workload”, suggesting longer waiting queue that might impair effective use of healthcare services [17, 18].

Regarding *retention in care*, facilities located in urban settings appear to have better performance as compared to those based in rural settings. Thus, to improve retention in care, there is need to distinguish and target adolescents who are lost to follow-up from those with voluntary treatment interruption. In the Namibian study, 82% of paediatric clinics attained the desired performance for retention in care with no significant difference between reference and outreached sites [20]. Poor performance on *retention in care* could be attributed to far distances to health facilities and high patient-to-staff ratio (i.e. higher workload) in rural settings. Of note, the problem of staff shortages could affect the recording system, which in turn may impair adherence monitoring [17, 18]. Of note, in rural sites, it was challenging to trace the files of some adolescents who were actively receiving therapy, especially with changes in the physical locations (a case study of the Mbalmayo district hospital). Limited access to patient data in these rural settings could also be explained by the lack of electronic storage systems, suggesting a high possibility of missing data or lost to follow-up due to the limitation of the systems in place [17, 18]. A much better approach to solve this issue will be the collection and storage of patient records in a well-protected electronic device, alongside staff training and considerations for the software device maintenance.

Regarding “*drug stock outs*”, poor performance was recorded in both urban and rural settings. Surprisingly, drug stock outs were significantly more frequent in urban as compared to rural hospital pharmacies. This observation is similar to that obtained by Billong et al. in Cameroon [21], Mutenda et al. in Namibia [20], and Dube et al. in South Africa [22]. Inversely, 96% of 929 ART clinics in Thailand had no event of drug stock outs [23]. This disparity shows that the drug supply machinery remains challenging in SSA, and this calls for SSA countries to learn from the example of non- African RLS for procurement and stock management. As previously observed in adult populations [17–24], ARV stock out is a key contributor to HIVDR emergence among adolescents living in both urban and rural communities of SSA.

Regarding *dispensing practices*, all the ART clinics attained the desirable performance of 100% good dispensing practices in both urban and rural settings, suggesting also good prescription practices from clinicians as per previous reports in the same country [12, 18, 19], in other SSA countries [23, 24], and in the WHO HIV drug resistance monitoring progress report of 2018 [17]. This is essentially due to the wide use of standard triple ART regimens and continuous training of healthcare providers [17–20].

Regarding *viral load coverage*, a desirable outcome was reported solely in facilities based in urban settings. This can be explained by the fact that viral load monitoring is readily available in urban sites whereas there is scarcity in remote, catchment or outreached facilities [18]. Of note, the WHO 2018 HIVDR progress report revealed that only 10% (3/31) of HIVDR focus countries attained the desired threshold for viral load coverage [17]. Thus, the advent of point-of-care viral load testing should improve coverage in rural settings by leveraging on integrated diagnostic assays [28, 29]. Systemic factors could also contribute to limiting access to viral load, such as poor prescription of viral load tests by clinicians, lack of reagents and/or lack of electronic records in several RLS [17].

Regarding *viral suppression*, an acceptable performance was reported only in 20% of the sites, an outcome similar to that reported by the WHO (29% desirable performance) [19]. Thus, a majority of countries still face issues with viral suppression, which is usually influenced by the poor viral load coverage as aforementioned [24–26]. When access to viral load testing is limited, there may be preferential selection of patients with advanced clinical disease conditions for conducting viral loads, thereby leading to selection bias and lowering the overall suppression rates.

Looking at the performance of “adequate switch to second line”, only one-third of the sites reached the desirable target for switching adolescents to second line therapy based on a confirmed virologic failure on first-line ART [17]. Workload does not seem to affect this performance. It therefore appears that poor access to viral load or limited awareness on the viral load algorithm prompt switch to second-line ART is based solely on clinical and/or immunological features.

This program evaluation was conducted within the frame of the project entitled *Evaluation of Treatment Response, Drug Resistance and HIV*-*1 Variability among Adolescents on First*- *and Second*-*Line Antiretroviral Therapy in Cameroon: The READY*-*Study* [27]. Advanced analysis from this study on 98 sequences out of 126 adolescents experiencing virologic failure (i.e. HIV viral load ≥ 1000 copies/ml) revealed an overall prevalence of HIVDR of 95.91%, with 95.91% NNRTI-resistance, 81.63% NRTI-resistance and 3.1% PI/r-resistance [28]. These findings underscore the need for systematic resistance testing in paediatric populations after an unsuppressed viral load as previously observed by Nasuuna et al. in a similar RLS [29]. Of note, in the latter study, only 23% of children who had a viral load above 1000 copies had a viral suppression after intensified adherence counselling (IAC) [29]. Furthermore, viral suppression rates appeared to be low among ART-treated children with virologic failure despite completion of the recommended three IAC sessions. Thus, meeting the third-90 among children/adolescents warrants innovative/adapted strategies for IAC, which take into account the psychosocial context, the educational level, and even gender disparities as they grow toward adulthood [29].

In order of priority, *pharmacy stock outs* recorded the poorest performance (very unsatisfactory), suggesting drug stock out as the leading cause of HIVDR among adolescents. This is followed by *on*-*time drug pick*-*up*, *viral load suppression*, *adequate switch to second*-*line ART* and *viral load coverage* whose performances were slightly higher but unsatisfactory. This constitutes the specific package of indicators that merit timely interventions to address HIVDR emergence among adolescents. In the frame of the aforementioned interventions, alleviating the workload would contribute in improving the performance for *on*-*time drug pickup, retention in care, and viral load coverage*.

### Study limitation

Data from urban facilities was statistically higher as compared to those from the rural settings. This weakens data comparability by geographical locations. As an exhaustive sampling of eligible participants was conducted, abstracted data reflect the real-life estimates of adolescents monitored on ART in urban and rural settings. Furthermore, inappropriate recording systems and incomplete patient files also limited data abstraction. Stratified data of PQIs between early (10–14 years) and advanced age (15–19 years) adolescents should be considered in future studies.

## Conclusion

In the population of adolescents receiving ART in Cameroon, *pharmacy stock outs* is the major driver of HIVDR emergence, followed by *delayed drug pick*-*up*, *poor viral load suppression*, *viral load coverage* and *inadequate switch to second*-*line ART*. Thus, urgent interventions on these PQIs, focusing on rural settings, would limit the emergence of HIVDR among adolescents. Furthermore, alleviating the workload might contribute to improve *on time drug pickup, retention in care, and viral load coverage*. In both urban and rural settings, dispensing practices of triple ART regimens are excellent.

## Data Availability

Data supporting the findings are fully available in the results, in the tables and figures of the manuscript.
